# Mammographic compression practices of force‐ and pressure‐standardisation protocol: A scoping review

**DOI:** 10.1002/jmrs.400

**Published:** 2020-05-18

**Authors:** Elizabeth Serwan, Donna Matthews, Josephine Davies, Minh Chau

**Affiliations:** ^1^ UniSA Allied Health & Human Performance University of South Australia Adelaide Australia; ^2^ Medical Imaging Department Flinders Medical Centre Bedford Park SA Australia

**Keywords:** breast, mammographer, mammography, review, screening

## Abstract

**Introduction:**

As an efficient, effective and moderately inexpensive modality, mammography has been implemented as a cancer screening tool and in diagnostic management. However, appropriate breast compression is necessary for optimal outcomes. Current key measures of compression force are subjective and variable, giving rise to the concept of a ‘personalised’ pressure‐standardisation protocol.

**Methods:**

A scoping review of the literature was performed using the Arksey and O’Malley framework to explore the existing force‐ and pressure‐standardisation protocols in clinical application. A comprehensive search strategy and standardised study selection and evaluation were completed. This synthesis of existing knowledge can lead to the implementation of mechanically standardised mammographic compression pressure as a feasible tailored approach to clinical practice. Four databases (PubMed, MEDLINE, Embase and Scopus) were searched from the databases’ inception to 13 December 2019 for relevant information, and eighteen articles were selected for analysis.

**Results:**

In addition to current protocol comparison, emerging key concepts include the reasoning behind standardisation, the benefits of improved diagnostic outcomes/decreased pain with negligible change in image quality and average glandular dose (AGD), and the recommendation of a 10kPa (approximate) pressure‐standardisation protocol. Research to date is largely based abroad (Netherlands), with a strong focus on screening practices. Consequently, several gaps in the current literature were identified as potential directions for future investigation.

**Conclusions:**

As a suggested mammographic guideline, compression pressures of approximately 10kPa aid in image acquisition reproducibility both within and between women; pain levels decrease, with minimal variations to breast thickness, AGD and image quality.

## Graphical abstract

Current methods of mammographic compression are subjective and variable. Research supports an alternate standardised compression protocol founded on pressure; this approach accounts for individual breast characteristics in a ‘personalised’ manner. A compression pressure of approximately 10kPa was found to decrease pain, with a negligible effect on breast thickness, average glandular dose, and resultant image quality.

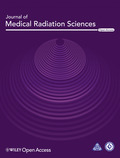

## Introduction

As breast cancer is one of the most prevalent female cancers, it is unsurprising that mammographic screening programs have been implemented worldwide.^1^ Such programs are estimated to result in an approximate annual reduction in breast cancer mortality by 30%.^2^ Additionally, mammography is a cost‐effective and gold standard practice.^3^ Sources report high sensitivity and the highest specificity in mammographic screening relative to MRI; higher MRI sensitivity does not translate into improved clinical outcomes due to the ineffective detection of ductal carcinoma in situ.^3^ Furthermore, mammography is the preliminary imaging modality used in most diagnostic settings (depending on patient age); calcium deposits, most frequently benign, can be indicative of potentially cancerous pathological change, which are usually seen clearly on a mammogram.^4^, ^5^ Breast compression is necessary for any successful mammographic examination; many sources discuss the effect of a uniform breast tissue thickness on improving image contrast and quality, with an associated reduction in radiation dose, geometric/motion blurring and tissue superimposition.^1^, ^5^, ^6^, ^7^, ^8^, ^9^, ^10^, ^11^, ^12^, ^13^, ^14^ Whilst ultimately aiding in the detection of pathology, the discomfort attributed to breast compression remains the highest reason for patient non‐compliance with screening programs and lack of diagnostic lesion visibility.^9^, ^15^, ^16^ According to the literature, the key factors affecting compression can be attributed to the patient, the mammographer and/or the equipment.^17^ However, compression standardisation is difficult due to the lack of explicit criteria to assess consistency; the predictability and reproducibility of mammographic examinations are thus difficult to determine.^13^ This is increasingly apparent when comparing the mammography protocols utilised on a global scale, in conjunction with international approaches to clinical practice – the specification of concrete parameters and/or guidelines is brief and minimal, with sufficient compression seemingly based on mammographer opinion and experience. The only objective measures currently obtainable in real time are those of compression force and breast thickness; these are gained mechanically from the mammography system, yet do not account for individual variations in breast size and elasticity.^13^ This has led to recent investigation of pressure (expressed in kilopascals – kPa) as a ‘personalised’ adaptation of force. Since pressure considers both the force and contact area simultaneously (pressure = compression force/breast contact area), it has been reported as a more physiologically appropriate compression parameter than force.^14^ In comparison, an opinion‐based application of force is variable and unreliable, whilst a mechanical application of target pressure is consistent and reproducible. Consequently, recent research has stemmed into developing an objective pressure‐standardisation protocol for clinical implementation, which allows for real‐time appraisal. Therefore, the aim of this review is to assess the feasibility of mechanically standardised mammographic compression in clinical practice. This will be evaluated in comparison with current techniques – both nationally and internationally – with the perceived benefits, recommendations and limitations outlined.

## Methods

### Method of review

A scoping, or ‘mapping’, review was performed to assess the current standing of mammographic compression practices available in existing literature. The organisational framework, derived as described by Arksey and O’Malley, was chosen as a means of evaluating the extent of available evidence for a clinical overview.^18^ As information from existing evidence was extracted and summarised for this scoping review, rather than methodologically appraised and statistically analysed, a systematic review was deemed unsuitable. To establish the context of this research in the wider mammographic community, the following research questions were pondered:

- Would mammographic practices benefit from mechanical standardisation of objective parameters?
- In what ways can compression force and compression pressure be justified as an appropriate measure of standardisation?
- How are outcomes relevant from both a clinical and patient‐centred perspective?

### Search strategy

Four electronic databases (PubMed, MEDLINE, Embase and Scopus) were searched for English‐language articles discussing mammographic compression practices from the databases’ inception to 13 December 2019; most sources were found to be relatively recent due to the nature of the topic. Structured search strategies were devised to optimise findings; the initial search began with the Medical Subject Heading (MeSH) term ‘mammography’, which was altered to ‘mammograph*’ as a database entry. Further keywords added to the search strategy included (AND) ‘compress*’ OR ‘force’ OR ‘pressure’, AND ‘standard*’. Although no relevant MeSH terms exist for such keywords, these were deemed relevant to the aims of the research. Search fields were set at ‘all fields’ for each database, except for Scopus, which was limited to ‘article title, abstract, keywords’. The number of hits from each database was 53, 35, 43 and 60 articles from Scopus, PubMed, MEDLINE and Embase, respectively.

Following the addition of hand‐sought records from expert contribution and duplicate removal by a single researcher, titles and abstracts were screened independently by two researchers. The independent screening and reviewing of eligible studies were in line with the 2005 scoping review framework by Arksey and O’Malley,^18^ as well as the Preferred Reporting Items for Systematic Review and Meta‐Analysis (PRISMA)^19^ guidelines. Specified eligibility criteria for each step of the selection process were adhered to systematically, with eligible articles then selected by reading the full text. Eligible titles were those relevant to ‘mammography’ or ‘breast’, and eligible abstracts discussed at least one aspect of ‘compression’, ‘force’ or ‘pressure’. Review articles, case reports, conference reports, letters, editorial comments, opinions (including qualitative reports) and non‐English articles were excluded. Other exclusion criteria included articles purely discussing quality assurance/physics without reference to clinical applicability. Any disagreement was discussed and resolved by consensus among the entire research team. The team comprised of researchers with a medical imaging background, including an experienced breast imaging specialist with post‐graduate qualification in breast imaging. The entire research team also had extensive experience in conducting scoping reviews, systematic reviews and meta‐analyses.

### Literature analysis

**Figure 1:**
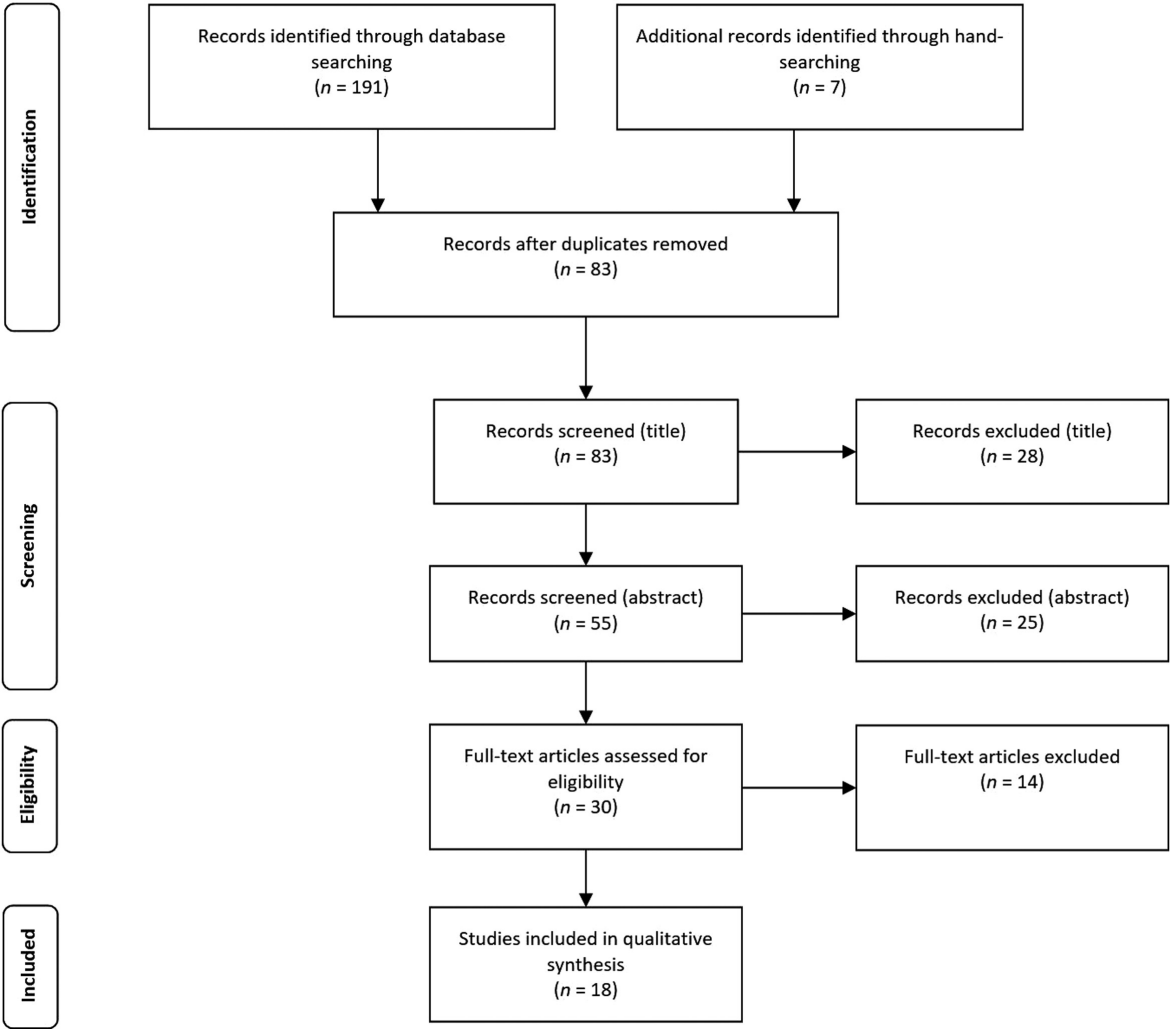
PRISMA flow diagram for scoping review data evaluation^19^

A data extraction form was developed using Microsoft Excel to collect information related to (i) country of origin and current practices, (ii) the perceived need for standardisation, (iii) benefits, (iv) recommendations and (v) limitations. Information compiled in the extraction form was reviewed by two researchers, with data analysed for key concepts and frequencies. These included overall benefits of the standardisation protocols, such as diagnostic outcomes, image quality, AGD and patient experience. A total of 18 articles were included for analysis in this scoping review; the PRISMA flow chart (Figure 1) details the review process, whilst Table 1 presents the summary of the reviewed studies, arranged alphabetically:

**Table 1:** This table presents the data extraction and analysis of the selected studies.

	Authors	Country of origin	Protocol used	Study details	Why standardise?	Benefits of pressure standardisation				**Recommendations**	**Limitations**
						Diagnostic outcome	Image quality	AGD	Patient experience		
1	Branderhorst et al.^13^	Netherlands (Netherlands vs USA)	**Netherlands**: Dutch screening QA protocol – 12daN. **USA:** no target – taut breast, unless painful	Retrospective cohort: **Netherlands**: screening asymptomatic patients, aged 50–75; CC + MLO **USA**: screening/diagnostic (symptomatic/ asymptomatic), aged 50–75; CC + MLO	Account for contact area; contact area decreases, average pressure/variance increase, despite lower force				Reduced pain	10kPa standard pressure; reduced variation and clear standards	Screening vs diagnostic cohort
2	Branderhorst et al.^20^	Netherlands		Prospective cohort: Real‐time and retrospective methods/software studied on asymptomatic patients; CC + MLO	Ensure contact area between breast/paddle can be determined for accurate pressure readings					Current systems give accurate/ precise pressure readings	Positioning discrepancies; diagnostic application
3	de Groot et al.^8^	Netherlands	Compress to 18daN or until pain threshold	Prospective observational: Hospital setting excluding interventions, patients aged 30–88; CC + MLO	Force‐controlled compression causes large pressure variation; pressure‐controlled accounts for contact area as predictive parameter for severe pain		Not compromised	Unchanged	Reduced pain	10–12kPa standard pressure; increased thickness of 9% (small) and 2% (large) breasts	Clinical only – not representative of screening population
4	de Groot et al.^21^	Netherlands	18daN compression protocol	Prospective observational: Real‐time examination of post‐intervention (unilateral) patients	Protocol applicable to post‐conservation therapy population				Reduced pain post‐intervention	Support for 10kPa standard pressure	Post‐intervention only
5	de Groot et al.^10^	Netherlands (comparing Netherlands with USA)	**Netherlands**: Dutch screening QA protocol – 12daN. **USA:** no target – taut breast, unless painful	Retrospective cohort: **Netherlands**: screening asymptomatic patients, aged 50–75; CC + MLO **USA**: screening/diagnostic (symptomatic/ asymptomatic), aged 50–75; CC + MLO	Account for contact area; contact area decreases, average pressure/variance increase, despite lower force		Potentially unchanged	Potentially unchanged	Potentially reduced pain	10kPa standard pressure	Screening vs diagnostic cohort
6	de Groot et al.^14^	Netherlands	14daN force standardisation, to be compared with 10kPa pressure standardisation	Prospective cohort: Screening asymptomatic patients aged 50–75; CC + MLO	Force applied in proportion to contact area (account for breast size)		Not compromised	Similar between protocols	Reduced pain: 1/2 cohort = less pain, 1/3 = unchanged	10kPa standard pressure (between venous/arterial blood pressure)	Screening only – not extend conclusions to lesion detection
7	de Groot et al.^5^	Netherlands	18daN force standardisation, compared to 10kPa standard pressure	Case–control: Case pairing of patients over time, with alternate protocol used for each; CC + MLO	Ensure lesion appearance is consistently satisfactory		Unchanged		Reduced unnecessary pain	Support for 10kPa standard pressure	Changes in practice between examinations
8	den Boer et al.^7^	Netherlands	Compress to 100–150N or until tolerance	Retrospective cohort: Screening follow‐up of asymptomatic patients, aged 36–74: CC + MLO (separate)	Compression as a function of contact area, to apply pressure independent of breast size		Not compromised, more consistent		Reduced pain	9.6–12kPa standard pressure	Time delay between successive acquisitions
9	Holland et al.^12^	Netherlands		Retrospective cohort: Screening asymptomatic patients, aged 50–75; MLO only	Possible improvement to screening program performance	Moderate pressures best (statistically insignificant)				Quantitative protocol; high compression worse on lesion visibility than low	No CC view
10	Holland et al.^6^	Netherlands		Retrospective cohort: Screening asymptomatic patients, aged 50–75; MLO views only	Possible improvement to screening program performance	High pressure decreases sensitivity; low pressure decreases specificity		Unchanged	Reduced pain	No optimal pressure; high compression worse on lesion visibility than low	No CC view; international screening differences; pectoral interference
11	Jeukens et al.^23^	Netherlands	10kPa standard compression	Retrospective cohort: Screening follow‐up of asymptomatic patients, mean age 59; CC view	Reduce pain due to compression			No clinically relevant difference	No clinically relevant difference	None of clinical relevance	Psychological impact on post‐intervention/ follow‐up examinations
12	Lau et al.^24^	Malaysia	Compress until taut or intolerable pain (no target force)	Retrospective cohort: Screening/diagnostic imaging of symptomatic/asymptomatic patients, aged 35–80 (clinical study); CC + MLO	Compression based on contact area; optimise protocols for Asian women (assumed to have smaller breasts)		Not compromised	Unchanged	Potentially reduced pain	Compression reduction of 32.5% from 12–9daN	Assume Asian women have small breasts, and study phantom = human tissue
13	Mercer et al.^17^	United Kingdom	Subjective mammographer judgement	Retrospective cohort: Evaluating mammographer practice in screening service	Minimise compression practice variance between/within mammographers					Focus on training process	Variation due to inconsistent examinations/patient modification
14	Mercer et al.^15^	United Kingdom	Subjective mammographer judgement	Retrospective cohort: Evaluating mammographer practice in screening service	Minimise compression practice variance between/within mammographers		Improved consistency	Improved consistency	Varied force impact screening attendance	Consistently applied force per patient, regardless of mammographer	Small, single‐centre study
15	Mercer et al^16^	United Kingdom	Subjective mammographer judgement	Retrospective cohort: Evaluating mammographer practice in screening service	Minimise compression practice variance between/within mammographers	Potential improvement in cancer detection	Potential positive impact	Potential reduction	Stabilisation may increase re‐attendance	Establish guidelines for cessation of compression force	Geographical proximity implies similar training
16	Moshina et al.^1^	Norway		Retrospective cohort: Screening asymptomatic patients aged 50–69; CC + MLO	Optimise the performance of screening measures	High force (proportional to pressure) decreases sensitivity and specificity	Possible improvement (inconclusive)			Force of at least 130N, pressure less than 9.8kPa	Patient positioning of questionable quality
17	Moshina et al.^22^	Norway	10kPa standard compression	Prospective cohort: Screening asymptomatic patients aged 50–69; CC + MLO	Minimise pain experienced during screening mammography		Reduced with pressure‐standardised paddle (inconclusive)	Increased with pressure‐standardised paddle	Higher pain with fixed paddle – clinically inconclusive	None of clinical relevance	Subjectivity of data acquisition
18	Poulos & McLean^9^	Australia	‘breast is taut at the sides'; 'skin blanches'	Prospective cohort: Based on participants in screening setting	More objective criteria for application of compression force					Compression practice focus: minimise breast thickness	

## Results

The retained articles consisted of a mixture of quantitative study methods. There were six prospective^8^, ^9^, ^14^, ^20^, ^21^, ^22^ and eleven retrospective studies,^1^, ^6^, ^7^, ^10^, ^12^, ^13^, ^15^, ^16^, ^17^, ^23^, ^24^ with one case–control study.^5^ Publication dates ranged from 2004 to 2019, with the last publication being a 2019 retrospective cohort study.^24^ Eleven studies were sourced from the Netherlands,^5^, ^6^, ^7^, ^8^, ^10^, ^12^, ^13^, ^14^, ^20^, ^21^, ^23^ with one remaining publication originating from Malaysia,^24^ two from Norway,^1^, ^22^ three from the United Kingdom^15^, ^16^, ^17^ and one from Australia.^9^ Twelve studies focussed on an asymptomatic screening population;^1^, ^6^, ^7^, ^9^, ^12^, ^14^, ^15^, ^16^, ^17^, ^20^, ^22^, ^23^ however, three studies investigated a diagnostic/post‐intervention population;^5^, ^8^, ^21^ and three studies considered both screening/diagnostic populations simultaneously.^10^, ^13^, ^24^ Twelve studies^1^, ^5^, ^7^, ^8^, ^10^, ^13^, ^14^, ^15^, ^16^, ^20^, ^22^, ^24^ investigated both standard mammographic views (cranio‐caudal (CC) and mediolateral oblique (MLO)), two studies investigated only the MLO view,^6^, ^12^ and one study investigated only the CC view.^23^ Two studies^22^, ^23^ were conducted from an equipment‐based perspective, focussing mainly on the compression paddle.

## Discussion of findings

This study aimed to provide a structured insight into the potential benefits of implementing mechanically standardised mammographic compression pressure in clinical practice. Findings are assessed in the following discussion alongside current practices and directions for suggested future research.

### Current practices

The subjectivity and lacking consistency of current compression guidelines are key concerns raised in much of the current literature; many studies describe the variation in applied compression force among mammographers, screening centres and countries.^1^, ^5^, ^6^, ^7^, ^8^, ^9^, ^10^, ^13^, ^14^, ^15^, ^16^, ^20^, ^24^ Clinically, force is typically adjusted in an experience‐based manner according to breast size, elasticity and pain threshold; ^8^, ^10^, ^13^, ^20^, ^21^, ^24^ Mercer et al. suggest that variation in compression may be associated with technique adaptation, as opposed to inconsistent practice.^17^ These parameters will differ significantly in a given population; thus, whilst conventional methods attempt to minimise disparity with the implementation of a target force, this is independent of individual breast characteristics.^6^, ^8^, ^13^ Further to this, it is noted that the compression forces used during mammographic examinations are attributed to the mammographer, as opposed to the patient.^15^, ^16^ Current European guidelines propose that ‘the breast should be properly compressed, but no more than is necessary to achieve a good image quality’;^11^ this corresponds with a maximum compressive force of 200N.^10^ Likewise, the United States of America (USA) recommendations suggest that the breast be compressed until ‘taut’ or ‘just less than painful’,^10^, ^13^ whilst studies conducted in Malaysia utilise similar subjective techniques.^24^ Dutch screening programs operate with a force compression protocol, with targets ranging between 12–18 daN.^5^, ^8^, ^10^, ^13^, ^14^, ^21^ A study completed by Branderhorst et al. comparing the mammographic compression practices within the USA and Netherlands found targets (mean ± standard deviation) to be 13.7 ± 5.9kPa versus 8.1 ± 4.1kPa, and 13.8 ± 2.7 daN versus 7.4 ± 3.1 daN for the Netherlands and USA, respectively.^11^ Overall, large variations in force, and even larger variations in pressure, were listed within and between both data sets, with distinctly higher compression limits in the Dutch data.^10^, ^13^ The variations in applied force and pressure during routine mammographic compression are supported with a phantom study completed by Lau et al..^24^ Regarding Australian practices, no formalised documentation of national protocols could be found in the literature, aside from stating the maximum operable force on a mammographic unit; this is 200N for motorised force and 300N for manual force.^25^ However, the subjective criteria of breast tautness and skin blanching are widely accepted.^9^ Overall, this seems to highlight a clinical need for standardised protocol.

### The need for standardisation

Pressure standardisation operates on the premise of compression force applied by the paddle to the breast, divided by the contact area between the paddle and the breast.^6^, ^7^, ^8^, ^13^, ^14^ By definition, it is most probable that pain experienced upon compression is more closely correlated with pressure than force.^6^ For completeness, Branderhorst et al. confirm that the contact area between the breast and compression paddle can be accurately and precisely determined with existing technology.^20^ Current literature suggests pressure is a ‘personalised’ version of force,^14^ with relevance to physiological factors such as tissue composition and blood pressure.^8^, ^14^ The theoretical dependence on contact area is of particular note; the same force results in a higher pressure when applied to a small contact area (i.e. small breast) than a large contact area (i.e. large breast).^1^, ^6^, ^7^, ^8^, ^10^, ^13^, ^14^, ^20^, ^21^, ^24^ The Dutch study conducted by Branderhorst et al. described a trend between force and contact area; average pressure and variance increases as contact area decreases.^13^ This was found to correlate with pain severity; patients with small breasts experience severe pain more often than large breasts when force standardisation is used.^14^ A Dutch study conducted by de Groot et al. reached similar conclusions; an intra‐individual comparison of 14daN force standardisation and 10kPa pressure standardisation was found to decrease pain severity without compromising image quality.^14^ A Norwegian study conducted by Moshina et al. extended these findings, concluding that pressure standardisation could optimise the performance of early screening measures.^1^ Ultimately, pressure‐standardisation protocols satisfy the need for objective criteria in mammographic breast compression; this would offer concrete mammographer guidelines, whilst consistently adjusting for individual breast parameters, such as size and elasticity.

### Benefits of pressure standardisation

The diagnostic outcomes of pressure‐standardised breast compression have been statistically evaluated in recent research. Generally, it was found that excessive pressure decreases mammographic sensitivity, whilst insufficient pressure decreases mammographic specificity.^6^ This reflects the findings described by Moshina et al., whereby a high compression force (directly proportional to pressure) decreased both sensitivity and specificity.^1^ The apparent conflict between the specificity outcome can be attributed to factors influencing resultant image contrast at the extreme ends of the compression scale. Also, Moshina et al. based conclusions on force alone, independent of contact area.^1^ This is important to note as results cannot be aligned with measures of compression pressure.

Current literature concludes that image quality remains unchanged with a pressure‐standardisation protocol;^5^, ^7^, ^8^, ^10^, ^14^, ^24^ no studies have shown otherwise. De Groot et al. investigated this trend explicitly, noting that although breast compression is milder with a pressure‐standardisation protocol, the visibility, contrast and sharpness of stable lesions remain virtually unchanged.^5^ Another study even proposed that a force reduction of as much as one third is possible with minimal impact on image quality.^24^ As noted by recent research, a large range of pressures result in diagnostically acceptable images in the digital setting;^10^, ^14^ a minimum must exist, though, as image quality is obviously degraded without compression. Nevertheless, Holland et al. suggest over‐compression may diminish image quality to a greater extent than under‐compression; lesion detectability is compromised as suspicious densities are dissolved.^6^ However, this primarily correlates with the high pressures required for spot compression. The opposite can likewise be reasoned; too little compression degrades image quality in that the potential for small cancers to remain undetected increases.^9^

The amount of radiation dose received by the patient with a shift in compression protocol was also investigated. Overall, it could be concluded that pressure standardisation has a negligible impact on AGD, despite the slight increase in breast thickness.^6^, ^8^, ^10^, ^14^, ^24^ Whilst the literature does demonstrate variation in data surrounding the actual dose received, this may possibly be attributed to different system types and automatic exposure control (AEC) settings.^13^ The proportion of image repeats was also found to remain unchanged.^14^

Although another recurring issue associated with breast compression is the patient experience of pain, a pressure‐based protocol was found to reduce unnecessary pain.^5^, ^6^, ^7^, ^10^, ^13^, ^14^, ^21^ This has the potential to positively impact compliance with screening programs^9^, ^16^, ^17^ and benefit the post‐intervention patients for whom follow‐up mammograms are mandatory.^21^ De Groot et al. revealed that the implementation of pressure standardisation resulted in half the cohort reporting less pain and a third reporting no difference when compared to force standardisation.^14^ However, this appears to apply to protocols in entirety; studies explicitly comparing force‐ and pressure‐controlled compression paddles found no preferential basis for either in clinical practice.^22^, ^23^ Moshina et al. suggest that pressure‐standardised paddles may reduce image quality and increase AGD,^22^ although this requires additional research beyond the scope of this review to validate.

### Recommendations

Current literature offers a pressure‐standardised compression protocol of approximately 10kPa, with some variation.^1^, ^7^, ^8^, ^10^, ^13^, ^14^, ^24^ Although many studies recorded higher average values in the actual results, this can be attributed to subjective force standardisation as the foundation of data collection. One study indicated positive outcomes occur with a pressure reduction from 12–9kPa,^24^ another proposed an ideal force of at least 130N with associated pressure less than 9.8kPa,^1^ another recommends a standard pressure between 9.6 and 12kPa,^7^ and yet others suggest 10kPa results in an ideal tissue pressure between that of normal venous and arterial blood pressure.^14^, ^21^ Research conducted by de Groot et al. similarly concluded that a compression of 10–12kPa corresponds with breast arterial pressure, although an increase in thickness of 9% for small and 2% for large breasts was also recorded.^8^ Whilst not ideal, this increase in thickness may be considered negligible due to the large dynamic range of digital mammography systems, as discussed previously. This notion is supported by Lau et al; a reduction in compression force of 32.5% has minimal effect on image quality and AGD.^24^ Low pressure and high breast volume are considered ideal for enhanced cancer detection,^1^ with pressures between 9.2 and 10.7kPa resulting in the highest detection rate.^10^, ^14^ Further to this, high compression has been shown to reduce lesion visibility to a greater extent than insufficient compression.^6^, ^10^, ^12^ Mercer et al. also evaluate this from a mammographer’s perspective, suggesting that extending mammographer training may aid performance and technique consistency.^15^, ^16^, ^17^ Another recommendation includes the need for peer observation to allow for comparison of a mammographer’s own force compression practice to that of colleagues.^26^

### Limitations

Several drawbacks arose when analysing the literature, many of which are applicable across multiple studies. Firstly, generalising conclusions to an external population is unwise, as screening policies and populations differ across countries.^6^ This also raises the issue of extending results between screening and diagnostic mammography; as suggested by the literature, a diagnostic or post‐intervention cohort may experience increased sensitivity to pain and hence are less tolerant to compression.^8^, ^10^, ^13^, ^14^, ^20^, ^21^ A study conducted by de Groot et al. supports this concept; the odds for severe pain in women post‐breast‐conserving therapy was 5.3 times higher than normal, largely due to changes in breast composition (elasticity).^21^ Accounting for differences between standard mammographic views (CC and MLO) is also important; the inherent discrepancies resulting from alternating the positioning technique limit the comparability of recorded values, both within and between studies.^6^, ^12^ Additionally, the impact of pectoral muscle inclusion in the MLO contact area was reported; due to its presence, the computed pressure does not accurately reflect the pressure exerted on the breast tissue.^6^ Clinically, the appearance of specific lesion types experiencing over‐ or under‐compression is inconclusive.^6^ Furthermore, variations across study types are useful to note; parameters available for acquisition and analysis differ depending on whether data collection was prospective or retrospective.

Limitations inherent in the review process itself must also be acknowledged. Timeframe limits were not applied, although dates were consciously noted throughout analysis; most studies were relatively recent regardless (i.e. all 2013 onwards, excluding two studies), given this field is currently evolving. Applying language‐limiting parameters resulted in the omission of non‐English sources; hence, scholarly evidence in foreign languages is not captured in this review. Upon analysis, most information originated from the Netherlands; this may demonstrate selection bias in that evidence for favoured outcomes is strengthened, yet similar conclusions were reached in the other literature. An extended search of more databases and ‘grey’ literature would confirm this. In fact, the distribution of resource origins serves to highlight the identified lack of Australian evidence, whilst reinforcing this review’s objective of pressure‐standardisation protocol in a national context. Although potentially indicative of publication bias, this could not be assessed as no accepted method exists for its evaluation of diagnostic test accuracy studies.^27^ Furthermore, the selection of sources may display potential subjectivity as only two reviewers were involved in the process; reproducible criteria were adhered to in order to minimise this. Similarly, it is possible that data were missed due to keyword specifications and terminology issues.

## Conclusion

The notion of mechanical standardisation techniques for breast compression is central to recent mammographic advances, with existing literature strongly supporting its implementation. Based on the current data available, several conclusions can be deduced. Key mammographic measures are obtained subjectively; the resultant disparity thus infers that optimal compression force values are not present in published research guidelines. Research supports an alternate standardised compression protocol founded on pressure; this approach accounts for individual breast characteristics in a ‘personalised’ manner.^7^, ^8^ As well as an objective measure, a compression pressure of approximately 10kPa was found to decrease pain, with a negligible effect on breast thickness, AGD and resultant image quality.^1^, ^6^, ^7^, ^8^, ^10^, ^13^, ^14^, ^24^ This also aids the reproducibility of image acquisition between and within women, whilst offering suggested guidelines for mammographers.^7^, ^10^, ^13^ It is therefore suggested that patient compliance would increase in accordance with the perceived benefits of a standardised technique, which would ultimately aid in the detection of early‐stage breast cancer.^9^, ^16^, ^17^ However, recent literature also presents several limitations which may ideally be considered before widespread clinical application. This is particularly relevant as current Australian data are lacking; hence, the generalisation of evidence‐based conclusions to a specific population requires additional research.

## Conflict of Interest

The authors declare no conflict of interest.
